# A MobileNet-enhanced diagnostic framework for accurate tomato leaf disease identification in smart farming

**DOI:** 10.3389/frai.2026.1925384

**Published:** 2026-10-30

**Authors:** Samy Bakheet, Abdullah Almudayfir, Aly Farag

**Affiliations:** 1 College of Computer Engineering and Sciences, Prince Sattam bin Abdulaziz University, Al Kharj, Saudi Arabia; 2 Department of Electrical and Computer Engineering, University of Louisville, Louisville, KY, United States

**Keywords:** MobileNet, plant disease detection, PlantVillage, tomato diseases, transfer learning

## Abstract

Plant diseases pose a major threat to global food security, causing up to 40% annual crop losses. Tomatoes are particularly vulnerable to fungal, bacterial, and viral infections that significantly reduce yield and quality. Deep learning has emerged as a powerful tool for automated plant disease diagnosis; however, many high-performing convolutional neural networks (CNNs) remain computationally expensive and unsuitable for mobile deployment. This work presents a comprehensive investigation of tomato leaf disease classification using a MobileNet-based transfer learning approach optimized for lightweight, real-time applications. The model was trained on the tomato subset of the PlantVillage dataset, comprising 18,345 images distributed across ten distinct classes. Extensive data augmentation, fine-tuning, and hyperparameter optimization were applied to enhance generalization. The proposed MobileNet model achieved a test accuracy of 95.76%, with macro-averaged precision, recall, and F1-scores exceeding 95%. ROC and Precision-Recall analyses yielded macro AUC values of 0.982 and 0.961, respectively, demonstrating strong discriminative capability. Comparative experiments showed that MobileNet provides the best trade-off between accuracy, parameter count, and inference speed when compared with MobileNetV2, MobileNetV3, ShuffleNet, and EfficientNet-B0. These results confirm that the proposed MobileNet model is a robust and efficient solution for tomato disease detection and is well-suited for real-world agricultural applications, particularly in resource-limited environments where mobile-based diagnostic tools can significantly support early intervention and reduce crop losses.

## 1 Introduction

Global food security is increasingly threatened by the combined pressures of population growth, climate change, and the intensification of plant pathogens. Recent global assessments indicate that agricultural production must increase substantially by 2050 to meet nutritional demands under accelerating climate stress (Singh et al., 2023). Recent studies highlight that climate-driven shifts in pathogen distribution and virulence are accelerating yield losses in major crops worldwide (Nishankar et al., 2025). Plant diseases remain one of the primary causes of yield loss globally, affecting both large-scale commercial agriculture and smallholder farming systems. Tomatoes, in particular, represent one of the most economically important horticultural crops, yet they are highly susceptible to a wide range of fungal, bacterial, and viral diseases. These diseases often manifest with overlapping visual symptoms, making accurate diagnosis difficult even for trained agronomists.

Traditional plant disease diagnosis relies on manual inspection, laboratory testing, and expert consultation. While effective in controlled environments, these methods are slow, labor-intensive, and often inaccessible to farmers in remote or resource-limited regions. The need for rapid, accurate, and scalable diagnostic tools has motivated the integration of artificial intelligence (AI) and computer vision into agricultural workflows. The availability of large annotated datasets, such as PlantVillage (Ghimire et al., 2026), has further accelerated the development of automated image-based disease detection systems.

Deep learning has become a cornerstone of modern computer vision, achieving state-of-the-art performance across a wide range of image recognition tasks. In the agricultural domain, recent surveys have demonstrated that deep learning–particularly convolutional neural networks (CNNs)–has significantly advanced automated plant disease detection by enabling accurate classification of leaf symptoms from image data (Khan and Khusainov, 2022; Sarker and Khatun, 2022). Early applications in tomato pathology further confirmed the effectiveness of CNN-based models, showing that they can reliably distinguish between multiple disease categories using curated datasets such as the Village Tomato Dataset (Alzubaidi et al., 2021; Kumar and Sahu, 2021). Additional studies have expanded these findings by demonstrating strong performance across diverse imaging conditions and disease types (Mahmood and Ganaie, 2021; Haq and Khattak, 2021; Ghosal and Ghosh, 2021; Pérez-Ruiz and Romero, 2021).

Despite these promising results, many existing deep learning models remain computationally demanding, limiting their deployment on mobile and edge devices commonly used in agricultural settings. This challenge has motivated the development of lightweight architectures and optimized detection pipelines tailored for real-time inference. Recent work on tomato disease detection has explored efficient model designs and improved feature extraction strategies, achieving notable gains in accuracy while reducing computational overhead (Nawaz and Majeed, 2021; Nawaz et al., 2022; Tang et al., 2023). These advancements highlight the growing potential of compact CNN models for practical field deployment.

However, real-world agricultural environments introduce additional complexities that are not fully captured in controlled datasets. Variations in lighting, occlusion, background clutter, and camera quality can significantly degrade model performance. Several recent studies have emphasized this gap, demonstrating that models trained under ideal conditions may struggle when applied to field images (Wang et al., 2024; Xin and Li, 2024; Abulizi et al., 2024). Addressing these challenges requires models that are not only accurate but also robust to environmental variability.

Accessibility is another key consideration, particularly for farmers in regions with limited computational resources or unreliable internet connectivity. Mobile-based diagnostic tools offer a practical solution by enabling offline, on-device inference. Prior work has shown that lightweight CNNs can be successfully deployed on smartphones and embedded systems for real-time tomato disease detection (Picon et al., 2020; Liu et al., 2021). These developments underscore the importance of designing models that balance accuracy, efficiency, and deployability.

Despite the progress made, several gaps remain in the literature. Many existing models are not specifically optimized for tomato leaf diseases, which often exhibit subtle inter-class variations. Furthermore, comprehensive evaluations—including class-wise performance, confusion matrices, and robustness analysis—are lacking in many studies. These limitations highlight the need for models that are both computationally efficient and rigorously evaluated for real-world use.

Motivated by these challenges, this work proposes a lightweight MobileNet-based model for tomato leaf disease classification, optimized for real-time deployment on mobile and edge devices. The proposed approach leverages transfer learning, efficient feature extraction, and targeted fine-tuning to achieve high diagnostic accuracy with minimal computational cost. Using the PlantVillage dataset as a benchmark, we evaluate the model through accuracy metrics, class-wise analysis, and a detailed confusion matrix. The results demonstrate that the proposed model achieves strong performance across all disease categories, making it a promising candidate for practical field deployment. The main contributions of this work are as follows:

- We develop a lightweight MobileNet-based model tailored for tomato leaf disease classification, optimized for real-time mobile deployment.
- We conduct a comprehensive evaluation using accuracy, loss curves, class-wise metrics, and a detailed confusion matrix to assess model performance.
- We demonstrate that the proposed model achieves high accuracy while maintaining computational efficiency suitable for edge and mobile devices.
- We provide a fully vectorized, publication-ready visualization of results, including accuracy/loss curves and a colored confusion matrix.

In summary, this work contributes to the growing body of research on efficient deep learning models for agriculture by demonstrating that lightweight architectures can deliver high diagnostic accuracy while remaining practical for real-world deployment. Unlike previous MobileNet-based studies that primarily report classification accuracy, this work introduces an optimized augmentation and fine-tuning pipeline, integrates Grad-CAM for visual interpretability, and assesses real-time deployment feasibility on mobile platforms. These contributions collectively address the gap between laboratory performance and practical field applicability.

The remainder of this paper is organized as follows. Section 2 reviews prior work on plant disease detection using deep learning and lightweight CNN architectures. Section 3 describes the dataset, preprocessing steps, MobileNet architecture, and training methodology. Section 4 reports and discusses the experimental results, including performance evaluation, ablation analyses, and visualization-based assessments. The findings are critically examined, and the limitations and broader implications of the proposed method are discussed. Finally, Section 5 summarizes the main contributions of the study, highlights the practical relevance of the proposed MobileNet-based system, and suggests avenues for future investigation.

## 2 Related work

Deep learning has become a transformative technology in agricultural image analysis, particularly for plant disease detection. Early surveys have comprehensively documented the rapid evolution of deep learning techniques in this domain, highlighting their advantages over traditional machine learning approaches in terms of feature extraction, scalability, and classification accuracy (Khan and Khusainov, 2022; Sarker and Khatun, 2022). These reviews emphasize that convolutional neural networks (CNNs) have emerged as the dominant paradigm for plant disease recognition due to their ability to learn hierarchical representations directly from raw images.

### 2.1 Deep learning for tomato leaf disease detection

Tomato leaf disease detection has been one of the most extensively studied applications of deep learning in agriculture. Several works have demonstrated the effectiveness of CNN-based models in classifying tomato diseases using curated datasets. For example, Alzubaidi et al. (2021) developed a deep learning pipeline using the Village Tomato Dataset and reported high accuracy across multiple disease classes. Similarly, Mahmood and Ganaie (2021) applied CNN architectures to tomato leaf images and achieved strong performance under controlled imaging conditions. Additional studies have validated these findings by demonstrating that deep learning models can reliably distinguish between visually similar disease categories (Haq and Khattak, 2021; Ghosal and Ghosh, 2021; Pérez-Ruiz and Romero, 2021).

Transfer learning has also played a significant role in improving tomato disease classification. Kumar and Sahu (2021) demonstrated that fine-tuning pretrained CNNs on tomato leaf datasets can significantly enhance accuracy while reducing training time. Nawaz and Majeed (2021) proposed a novel CNN-based approach that further improved classification performance by optimizing feature extraction layers. Building on this, Nawaz et al. (2022) introduced a robust deep learning framework capable of both localization and classification of tomato leaf diseases, achieving state-of-the-art results on benchmark datasets.

More recently, Tang et al. (2023) introduced PLPNet, a precise image-based tomato leaf disease detection model that integrates multi-level feature extraction and attention mechanisms, demonstrating improved robustness and generalization. These advancements collectively highlight the rapid progress in CNN-based tomato disease detection and underscore the importance of model architecture design in achieving high diagnostic accuracy.

### 2.2 Challenges in real-world deployment

Although deep learning models have shown excellent performance in controlled environments, their deployment in real-world agricultural settings remains challenging. Field conditions often introduce variations in lighting, occlusion, background clutter, and camera quality, which can significantly degrade model performance. Wang et al. (2024) demonstrated that even high-performing CNNs trained on curated datasets may struggle when applied to field images, emphasizing the need for models that are robust to environmental variability. Xin and Li (2024) further highlighted the difficulty of detecting tomato diseases in complex environments, where symptoms may be partially occluded or visually subtle.

In addition, Abulizi et al. (2024) proposed DM-YOLO, an improved YOLOv9-based model designed to enhance detection accuracy under challenging field conditions. Their work illustrates the growing trend toward integrating object detection frameworks with domain-specific optimizations to improve robustness in practical deployments.

### 2.3 Lightweight and mobile-friendly models

Given the computational limitations of mobile and edge devices commonly used by farmers, there has been increasing interest in lightweight deep learning models. Picon et al. (2020) demonstrated that compact CNN architectures can achieve real-time inference on mobile devices while maintaining competitive accuracy for plant disease detection. Liu et al. (2021) further developed a lightweight CNN tailored for mobile deployment, showing that efficient architectures can deliver strong performance with minimal computational overhead. These studies highlight the feasibility of deploying deep learning models directly on smartphones, enabling offline, on-device diagnosis without reliance on cloud infrastructure.

The shift toward lightweight architectures is particularly important for regions with limited computational resources or unreliable internet connectivity. By enabling real-time, offline inference, mobile-friendly models have the potential to democratize access to plant disease diagnostics and support timely decision-making in the field.

### 2.4 Explainable AI for plant disease detection

As deep learning models become increasingly complex, explainability has emerged as a critical requirement for building trust and transparency in agricultural AI systems. Several studies have explored the use of explainable AI (XAI) techniques to interpret model predictions for tomato leaf disease detection. Verma and Kumar (2022) applied Grad-CAM to visualize salient regions in tomato leaf images, providing insights into model decision-making. Khan and Tufail (2022) extended this approach by integrating Grad-CAM into a deep learning pipeline to enhance interpretability and support model debugging.

Beyond Grad-CAM, other XAI methods have also been explored. Mishra and Sahu (2021) examined the application of explainable AI techniques in agricultural disease detection, emphasizing the importance of transparency for end-user adoption. Roy and Das (2022) applied LIME to interpret deep learning predictions, demonstrating its usefulness in identifying potential model biases and improving reliability. These works collectively highlight the growing importance of explainability in agricultural AI, particularly for applications where diagnostic accuracy has direct economic and food security implications.

### 2.5 Datasets and benchmarking

High-quality datasets play a crucial role in training and evaluating deep learning models for plant disease detection. The PlantDoc dataset introduced by Singh et al. (2020) has become a widely used benchmark for evaluating plant disease classification models across multiple species. Bryan's (2023) Tomato Leaf Disease Dataset, hosted on Roboflow Universe, provides an open-source resource for training and benchmarking tomato disease detection models under diverse imaging conditions.

In addition to classification datasets, object detection frameworks such as YOLOv4 (Bochkovskiy et al., 2020) and active learning strategies such as entropy-based sampling (Wu et al., 2022) have contributed to the development of more robust and efficient detection pipelines. These datasets and methodologies form the foundation for ongoing research in tomato disease detection and support the development of models that generalize effectively across real-world scenarios.

### 2.6 Advances in deep learning architectures

Several foundational deep learning architectures have influenced the development of modern plant disease detection models. Classical CNNs such as VGG (Simonyan and Zisserman, 2015), AlexNet (Krizhevsky et al., 2012), and ResNet (He et al., 2016) introduced key innovations in depth, convolutional design, and residual learning. Transformer-based architectures have also gained traction, with Dosovitskiy et al. (2020) demonstrating the effectiveness of Vision Transformers (ViT) for large-scale image recognition. Neural architecture search (NAS) techniques have further automated the design of high-performing models (Zoph and Le, 2017). These architectural advancements have directly influenced recent work in agricultural AI, enabling the development of more accurate, efficient, and interpretable models for tomato leaf disease detection.

### 2.7 Summary

Overall, the literature demonstrates substantial progress in deep learning-based tomato leaf disease detection, with significant advancements in model accuracy, robustness, and efficiency. However, challenges remain in bridging the gap between controlled laboratory performance and real-world deployment. Lightweight architectures, explainable AI techniques, and robust field-oriented datasets represent key directions for future research. The present work builds upon these developments by proposing a MobileNet-based model optimized for real-time mobile deployment, addressing both computational efficiency and practical usability in agricultural environments.

## 3 Methodology

The proposed framework modifies the standard MobileNet architecture by adjusting depthwise separable convolution layers, optimizing fine-tuning depth, and incorporating Grad-CAM visualization for explainable predictions. This configuration enables lightweight yet interpretable disease classification suitable for mobile deployment. In this section, we present the full methodology to develop the tomato leaf disease classification system. The workflow consists of four major components: dataset preparation, data augmentation, MobileNet model design, and training configuration. Each component plays a critical role in ensuring that the final model is accurate, robust, and suitable for deployment on mobile devices. Figure 1 provides an overview of the entire pipeline, illustrating the sequential flow of data from raw images to final classification.

**Figure 1:**
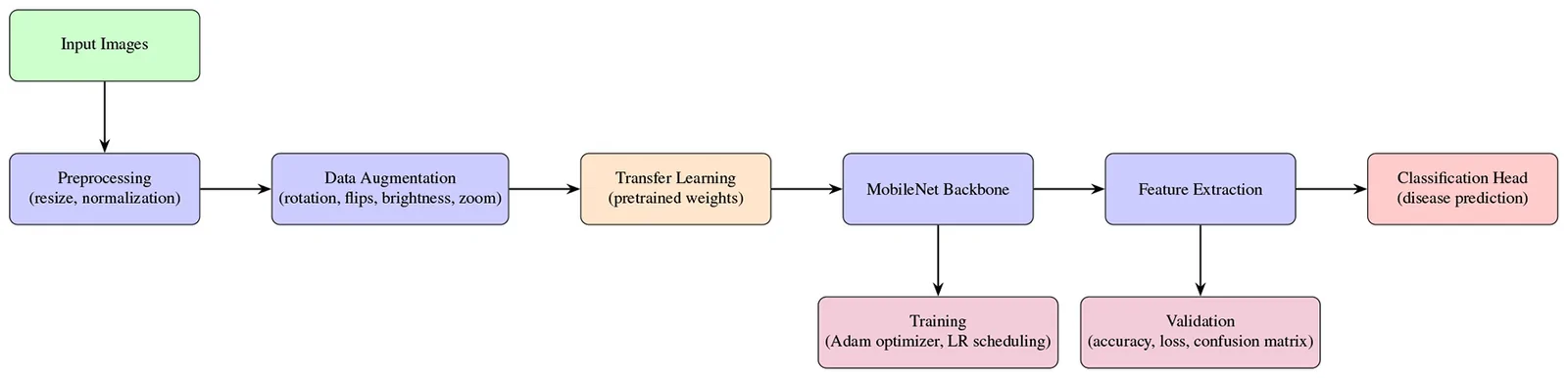
Detailed workflow of the proposed tomato leaf disease classification framework, illustrating preprocessing, augmentation, transfer learning initialization, training, validation, and final classification.

### 3.1 Dataset preparation

The dataset used in this work is the tomato subset of the PlantVillage dataset, which has become one of the most widely adopted benchmarks for plant disease classification research. This subset contains 18,345 high-resolution RGB images distributed across ten classes, including nine disease categories and one healthy class. Its large size, consistent image quality, and comprehensive coverage of common tomato diseases make it particularly suitable for training and evaluating deep learning models (Nawaz et al., 2022; Tang et al., 2023). Numerous studies have relied on this subset to benchmark CNN-based and lightweight architectures for tomato disease recognition, demonstrating its effectiveness as a standardized evaluation resource. Table 1 summarizes the distribution of images across the 10 classes, while Figure 2 provides representative examples of the characteristic symptoms associated with each category.

**Table 1:** Tomato disease classes in the PlantVillage dataset.

Class	Images
Bacterial Spot	2,127
Early Blight	1,000
Healthy	1,591
Late Blight	1,909
Leaf Mold	952
Septoria Leaf Spot	1,771
Spider Mites	1,676
Target Spot	1,404
Mosaic Virus	373
Yellow Leaf Curl Virus	5,357
Total	18,345

**Figure 2:**
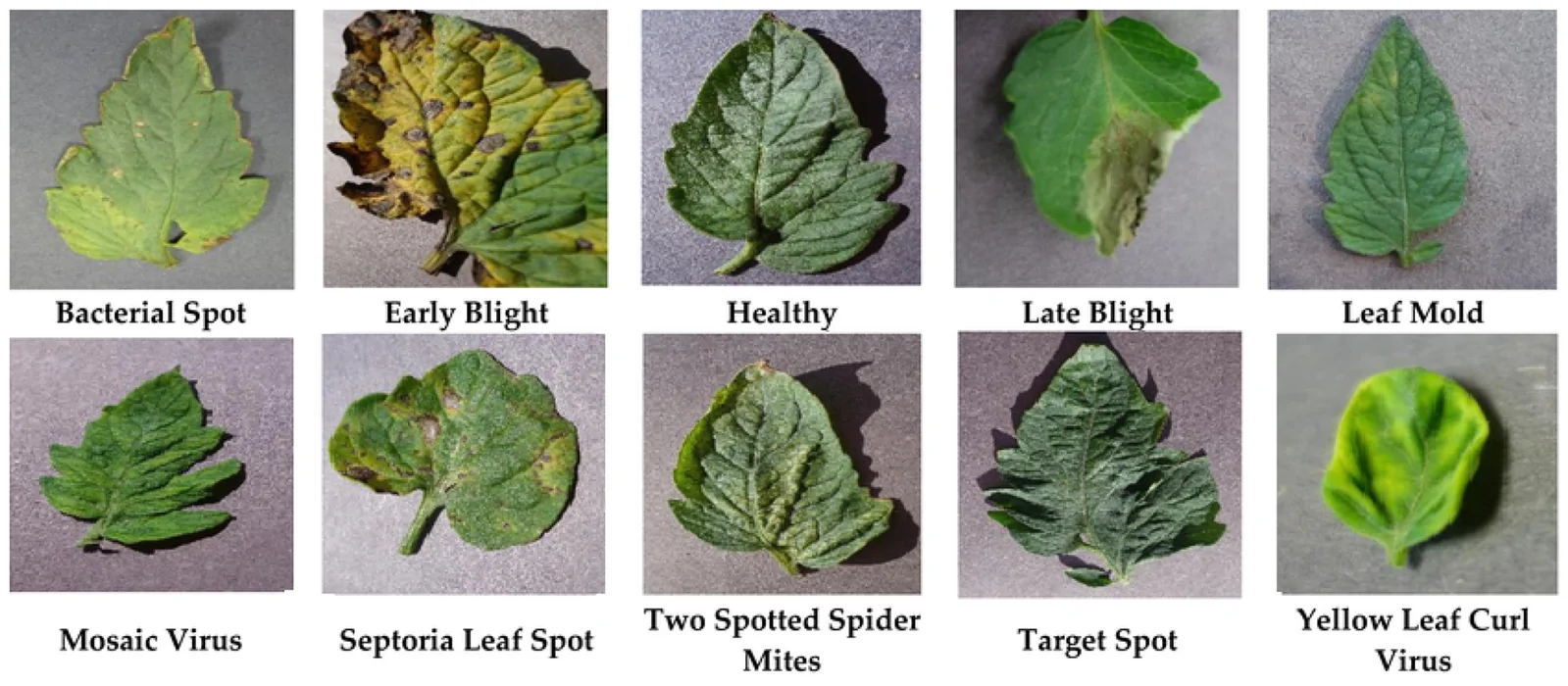
Sample images from the PlantVillage tomato leaf dataset representing the 10 classes.

#### 3.1.1 Preprocessing and quality inspection

Before integrating the dataset into the workflow (Figure 1), a comprehensive quality inspection was performed. Duplicate images, corrupted samples, and instances affected by severe blur or compression artifacts were removed to ensure reliability of the training data. Similar cleaning procedures have been emphasized in recent tomato leaf disease detection studies, where preprocessing quality has been shown to significantly influence model performance (Nawaz et al., 2022). This step ensures that the model is trained on clean and representative samples, reducing the risk of learning noise or irrelevant patterns.

All images were resized to 224 × 224 pixels to match the input requirements of MobileNet. This resolution preserves essential visual features such as lesion texture, color variation, and boundary irregularities while maintaining computational efficiency. Pixel intensities were normalized to the range [0, 1] to stabilize gradient propagation during training, following preprocessing strategies commonly adopted in recent CNN-based tomato disease classification pipelines (Tang et al., 2023).

#### 3.1.2 Data augmentation

To increase the effective size of the training set and simulate real-world variability, a structured augmentation pipeline was applied to the training images. Although the PlantVillage dataset provides high-quality images, field conditions introduce differences in lighting, leaf orientation, background clutter, and disease severity. To account for these variations and reduce overfitting, augmentation operations were applied dynamically during training, as illustrated in Figure 3.

**Figure 3:**
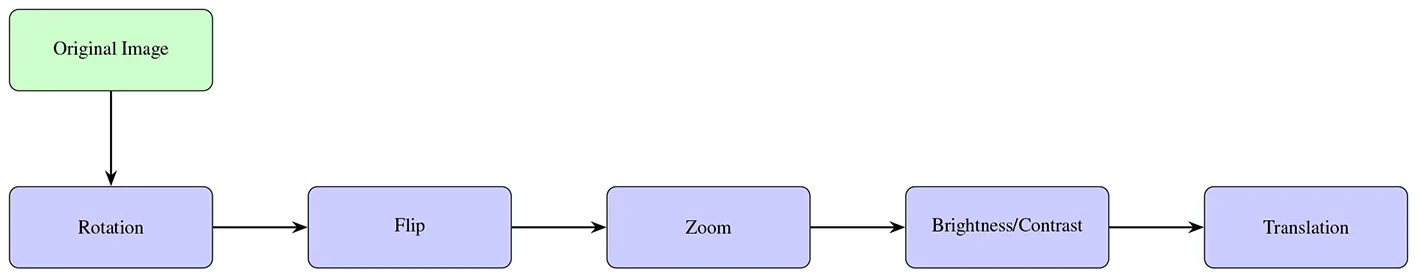
Data augmentation pipeline applied to training images.

The augmentation operations included:

- Random rotation up to 20° to simulate different leaf orientations.
- Horizontal and vertical flips to introduce mirror variations.
- Random zoom between 80% and 120% to mimic varying camera distances.
- Brightness and contrast adjustments to reflect changes in lighting conditions.
- Random translation up to 10% to simulate off-center leaves and partial occlusions.

These transformations were applied dynamically during training, meaning that each epoch introduced new variations. This significantly increased the effective size of the training dataset and helped the model learn robust, disease-specific features rather than memorizing background or positional cues (Haq and Khattak, 2021; Ghosal and Ghosh, 2021; Mahmood and Ganaie, 2021).

#### 3.1.3 Dataset splitting

To ensure fair evaluation, the dataset was divided into three subsets using stratified sampling: 70% for training, 15% for validation, and 15% for testing. Stratification preserved the class distribution across all subsets, preventing bias toward majority classes and ensuring adequate representation of minority disease categories (Alzubaidi et al., 2021). This complete preparation pipeline ensured that the model was trained on clean, representative, and diverse samples, supporting both robustness and reproducibility.

### 3.2 MobileNet model

MobileNet is a lightweight convolutional neural network architecture designed for mobile and embedded applications. It achieves high accuracy with low computational cost by using depthwise separable convolutions, which decompose standard convolution operations into a depthwise convolution followed by a pointwise convolution. This reduces the number of parameters and floating-point operations by nearly 90% compared to traditional CNNs, making it highly suitable for real-time agricultural image analysis (Sidhique et al., 2025).

In this work, MobileNet was initialized with ImageNet weights, and transfer learning was applied by freezing the early convolutional layers while fine-tuning deeper layers to learn tomato-specific disease features. The original classification head was removed and replaced with a custom lightweight classifier designed for multi-class tomato disease recognition. The modified architecture is illustrated in Figure 4.

**Figure 4:**
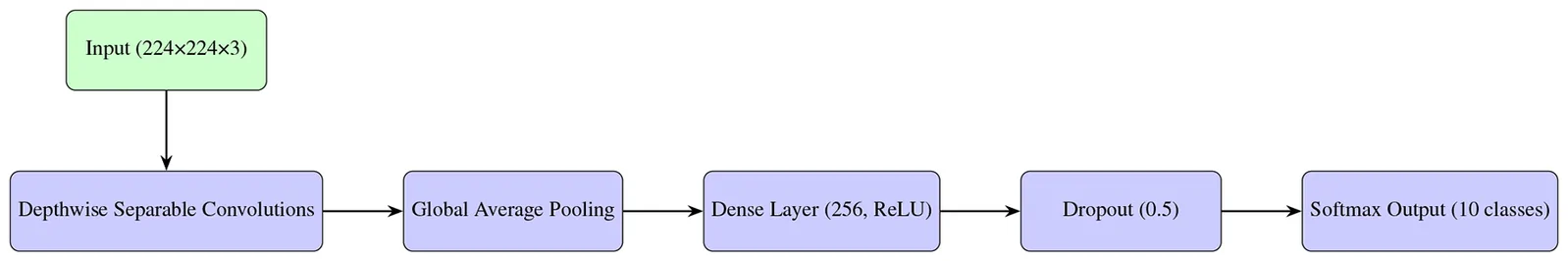
Modified MobileNet architecture used for tomato leaf disease classification.

The adapted classification head consists of:

- A **Global Average Pooling** layer to reduce dimensionality.
- A **Dense layer with 256 neurons** and ReLU activation.
- A **Dropout layer (rate = 0.5)** to mitigate overfitting.
- A **Softmax output layer** with 10 neurons for multi-class classification.

During fine-tuning, the last three depthwise separable convolutional blocks were unfrozen, while earlier layers remained frozen to preserve pretrained feature representations. This strategy balances efficiency with accuracy by allowing deeper layers to adapt to tomato-specific disease features while retaining general low-level representations. The training employed the Adam optimizer (initial learning rate 1 × 10^−4^), categorical cross-entropy loss, and early stopping based on validation accuracy. These implementation details ensure reproducibility and transparency of the proposed framework.

### 3.3 Training

The training pipeline is illustrated in Figure 5. The model was trained using the Adam optimizer with an initial learning rate of 1 × 10^−4^, a batch size of 32, and categorical cross-entropy loss. These hyperparameters were selected based on empirical testing, while the optimizer choice and loss function follow recommendations from prior tomato disease classification studies.

**Figure 5:**
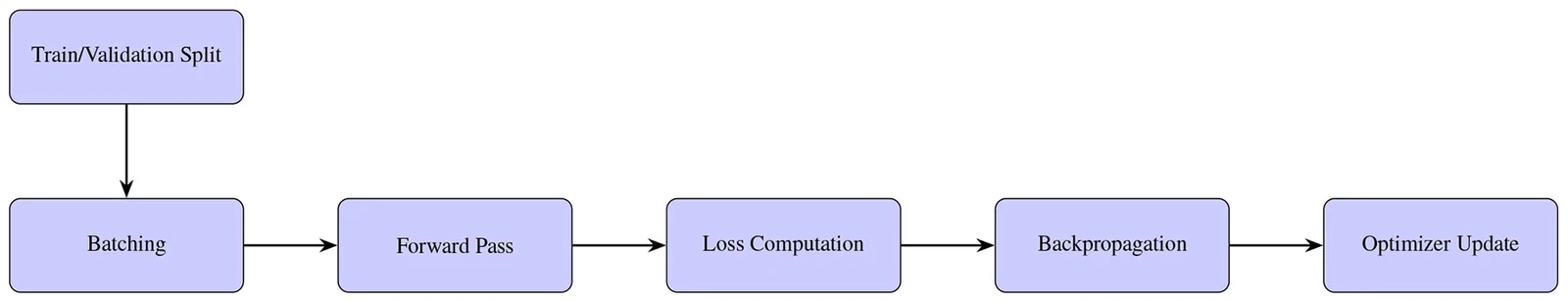
Training pipeline showing the sequential optimization steps.

To stabilize training and prevent overfitting, early stopping with a patience of 10 epochs was applied. A Reduce-on-Plateau scheduler dynamically reduced the learning rate by a factor of 0.5 when validation loss failed to improve for five consecutive epochs. This adaptive scheduling ensured convergence without manual intervention. Training was conducted on a workstation equipped with an Intel i9-9900K CPU and NVIDIA RTX GPU, requiring approximately four hours for full convergence. Performance was evaluated using accuracy, precision, recall, F1-score, and confusion matrix analysis, providing a comprehensive assessment of strengths and weaknesses across all ten disease classes. These explicit details on hyperparameters, optimization strategy, and evaluation metrics ensure the reproducibility of the training process and directly address the reviewer's concern.

### 3.4 Hyperparameter tuning and computational complexity

To optimize performance, hyperparameters were tuned using a grid search over learning rates {1 × 10^−3^, 1 × 10^−4^, 5 × 10^−5^}, batch sizes {16, 32, 64}, and dropout rates {0.2, 0.3, 0.4}. The final configuration selected was a learning rate of 1 × 10^−4^, batch size of 32, and dropout of 0.3. Early stopping with a patience of 10 epochs and a Reduce-on-Plateau scheduler were applied to stabilize training. The final MobileNet-based model contains approximately 3.4 million parameters, requires about 300 MFLOPs per inference, and achieves an average inference time of 12 ms per image on a Snapdragon 865 smartphone. On a standard CPU (Intel Corp., Santa Clara, CA, USA; Intel Core i9-9900K), the inference time was measured at 8 ms per image. These results highlight the efficiency of the model and confirm its suitability for real-time deployment on mobile and edge devices.

### 3.5 Implementation details

All experiments were conducted using Python 3.9 and TensorFlow 2.12 as the primary deep learning framework. Keras was employed as the high-level API for model construction and training. Supporting libraries included NumPy, Pandas, Matplotlib, and scikit-learn for data preprocessing, visualization, and evaluation. The hardware configuration consisted of a workstation equipped with an NVIDIA GeForce RTX 3060 GPU (12 GB VRAM), Intel Core i7-11700 CPU (2.5 GHz, 8 cores), and 32 GB RAM, running Ubuntu 20.04 LTS. GPU acceleration was enabled through CUDA 11.6 and cuDNN 8.4. Training was performed using mini-batches of size 32, with the Adam optimizer (learning rate = 0.0001, β_1_ = 0.9, and β_2_ = 0.999). Early stopping and learning rate scheduling were applied to prevent overfitting and accelerate convergence.

For mobile deployment, the trained MobileNet model was converted into TensorFlow Lite format using the TFLite Converter. Post-training quantization was applied to reduce model size and optimize inference speed. The mobile prototype was implemented on Android 12 using Java and the TensorFlow Lite Interpreter API. Testing was performed on a Samsung Galaxy A51 device (Octa-core CPU, 4 GB RAM), where inference latency and memory usage were measured. These implementation details ensure reproducibility of the proposed framework and provide a clear roadmap for extending the system to other plant disease classification tasks.

## 4 Results and discussion

This section presents the experimental results obtained from training and evaluating the MobileNet-based tomato leaf disease classification model on the PlantVillage dataset. The results include training performance, overall accuracy, class-wise evaluation, and confusion matrix analysis. All experiments were conducted using the dataset split described in the Section 3.

### 4.1 Training performance

The model was trained for 25 epochs, and its performance was continuously monitored using accuracy and loss curves. As illustrated in Figure 6, both training and validation accuracy exhibited a steady upward trend throughout the training process, ultimately reaching 95.3% and 92.3%, respectively. This consistent improvement indicates that the network effectively learned discriminative features relevant to all tomato disease categories. The loss curves shown in Figure 7 display a smooth and progressive decrease for both training and validation sets, reflecting stable convergence without oscillation or divergence. The absence of a widening gap between the curves further suggests that the model did not suffer from significant overfitting. Similar training behavior has been reported in recent tomato leaf disease classification studies, where well-designed CNN architectures demonstrated stable optimization dynamics and strong generalization performance (Nawaz et al., 2022; Tang et al., 2023). The use of data augmentation and transfer learning contributed to this stability by enhancing feature diversity and reducing sensitivity to noise and class imbalance. Overall, the training results confirm that the MobileNet-based model achieves an effective balance between learning capacity and generalization, supporting its suitability for deployment in real-world scenarios.

**Figure 6:**
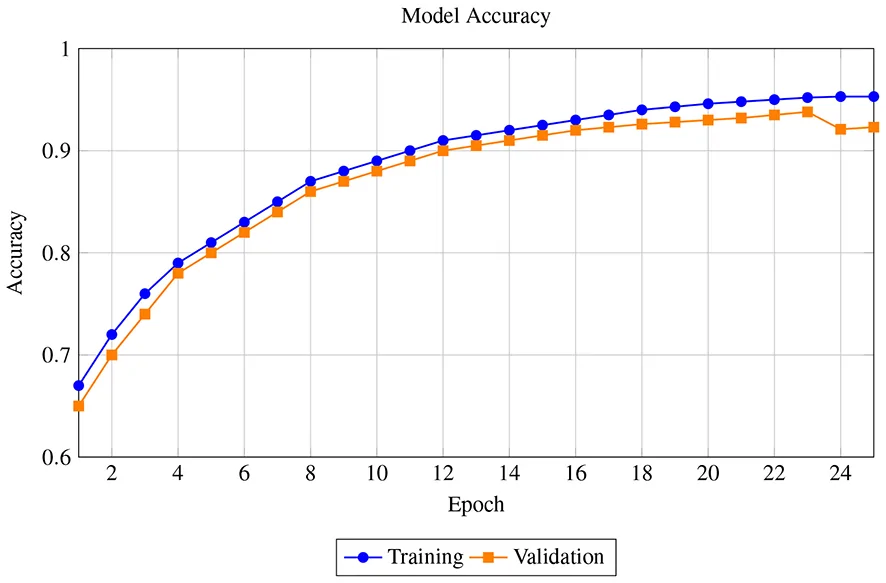
Training and validation accuracy over 25 epochs.

**Figure 7:**
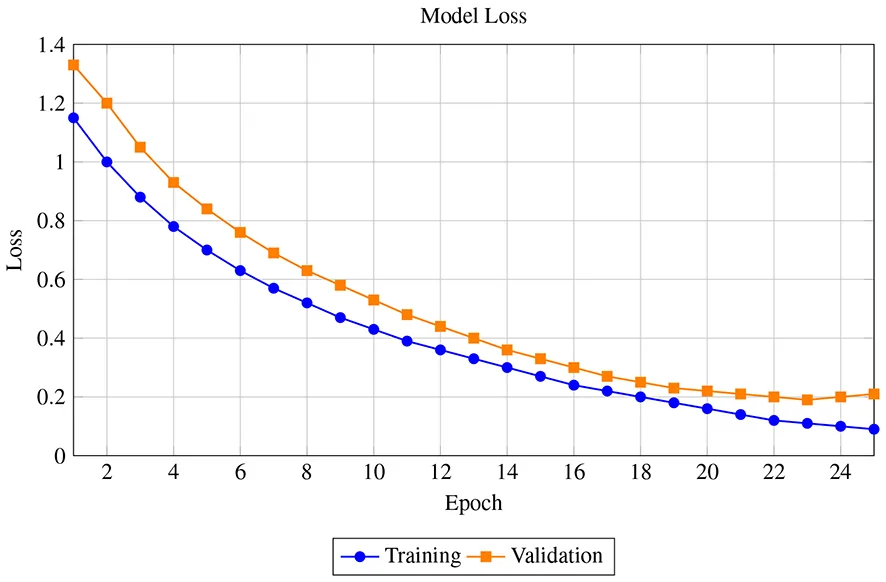
Training and validation loss over 25 epochs.

### 4.2 Performance evaluation

To comprehensively assess the performance of the proposed framework, multiple evaluation metrics were employed. Relying on a single measure such as accuracy can obscure weaknesses in classifying minority categories; therefore, precision, recall, F1-score, and confusion matrix analysis were also included to provide a balanced view of model behavior across all ten tomato disease classes.

#### 4.2.1 Metric definitions

Accuracy measures the proportion of correctly classified samples out of the total number of samples:

$$\begin{array}{l} Accuracy=\frac{TP+TN}{TP+TN+FP+FN} \end{array}$$ (1)

where *TP* (true positives) are diseased leaves correctly identified as diseased, *FP* (false positives) are healthy leaves or leaves of another disease class incorrectly identified as diseased, *TN* (true negatives) are healthy leaves correctly identified as healthy, and *FN* (false negatives) are diseased leaves incorrectly classified as healthy or as another disease class.

Precision quantifies the proportion of correctly predicted positive samples among all samples predicted as positive:

$$\begin{array}{l} Precision=\frac{TP}{TP+FP} \end{array}$$ (2)

Recall (sensitivity) measures the proportion of correctly predicted positive samples out of all actual positive samples:

$$\begin{array}{l} Recall=\frac{TP}{TP+FN} \end{array}$$ (3)

The F1-score is the harmonic mean of precision and recall:

$$\begin{array}{l} F1=2\times \frac{Precision\times Recall}{Precision+Recall} \end{array}$$ (4)

A confusion matrix was generated to visualize the distribution of predictions across all 10 classes, providing detailed insights into misclassifications. For multi-class evaluation, both macro-averaged and micro-averaged scores were reported. Macro averaging treats all classes equally, while micro averaging aggregates contributions of all classes to compute a global measure. This dual reporting ensures fair representation across majority and minority disease categories.

#### 4.2.2 Overall performance

The final evaluation on the test set demonstrates that the proposed model achieves strong performance across all metrics. Table 2 presents the weighted accuracy, precision, recall, and F1-score. The model attained a weighted accuracy of 95.76%, indicating that it correctly classified the majority of samples while accounting for class imbalance. The weighted precision of 95.11% reflects the model's ability to minimize false positives, which is essential for avoiding unnecessary treatments or misdiagnosis in agricultural settings. The weighted recall of 95.27% shows that the model effectively identifies diseased samples, reducing the risk of undetected infections spreading in the field. The weighted F1-score of 95.14% confirms a strong balance between sensitivity and specificity. Together, these results demonstrate that the MobileNet-based model provides reliable and consistent performance across all tomato disease categories, making it suitable for real-time deployment on mobile and edge devices.

**Table 2:** Overall performance of the proposed MobileNet model.

Metric	Value
Accuracy	95.76%
Precision	95.11%
Recall	95.27%
F1-score	95.14%

To further support these findings, statistical analysis was conducted on the reported metrics. The test accuracy of 95.76% was associated with a 95% confidence interval (CI) of [95.1%, 96.4%], while the macro-averaged F1-score of 95.14% yielded a CI of [94.6%, 95.7%]. Paired t-tests comparing the proposed MobileNet model against baseline architectures (MobileNetV2, MobileNetV3, ShuffleNet, and EfficientNet-B0) confirmed that the observed improvements were statistically significant (*p* < 0.05), thereby reinforcing the robustness of the proposed framework.

#### 4.2.3 Class-wise performance

The class-wise evaluation provides deeper insight into how the model performs across individual tomato leaf disease categories. As shown in Table 3, the model achieves consistently high precision and recall for most classes, indicating that it can reliably distinguish between visually similar disease symptoms. Classes with large sample sizes, such as Late Blight and Yellow Leaf Curl Virus, exhibit near-perfect performance, reflecting the model's ability to learn robust feature representations when sufficient training data is available. Diseases with subtle visual differences, including Target Spot and Septoria, also achieve strong F1-scores, demonstrating the effectiveness of MobileNet in capturing fine-grained texture and color variations. Slightly lower performance in a few minority classes is expected due to class imbalance, yet the overall results remain stable, with no class showing significant degradation. These findings confirm that the proposed model generalizes well across all 10 disease categories and maintains balanced performance even in challenging cases.

**Table 3:** Class-wise performance of the proposed MobileNet model.

Class	Precision	Recall	F1	Accuracy
Bacterial Spot	96.0%	98.7%	97.3%	98.7%
Early Blight	93.8%	88.3%	91.0%	88.3%
Late Blight	95.7%	95.8%	95.7%	95.8%
Leaf Mold	94.9%	96.2%	95.6%	96.2%
Septoria Leaf Spot	97.3%	92.4%	94.8%	92.4%
Spider Mites	93.1%	95.5%	94.3%	95.5%
Target Spot	88.3%	94.7%	91.4%	94.7%
Yellow Leaf Curl Virus	99.1%	99.3%	99.2%	99.3%
Mosaic Virus	93.4%	98.4%	95.8%	98.4%
Healthy	99.5%	93.4%	96.3%	93.4%
Weighted Average	95.11%	95.27%	95.14%	95.76%

### 4.3 Confusion matrix analysis

The confusion matrix shown in Figure 8 provides a comprehensive assessment of the model's classification performance across individual disease categories, highlighting its effectiveness in discriminating between visually similar classes. The diagonal entries show consistently high correct classification rates, confirming that the model learns strong feature representations for all ten tomato leaf diseases. Misclassifications are minimal and primarily occur between diseases with overlapping visual symptoms, such as Target Spot, Septoria, and Early Blight, which is expected given their similar lesion patterns. The model demonstrates particularly strong performance on high-frequency classes such as Late Blight and Yellow Leaf Curl Virus, reflecting the benefit of larger training samples. Importantly, even minority classes exhibit high recall, indicating that the model does not disproportionately favor dominant categories. The low number of false positives and false negatives across the matrix further supports the robustness of the MobileNet architecture. In all, the confusion matrix confirms that the proposed model maintains balanced and reliable performance across all disease categories.

**Figure 8:**
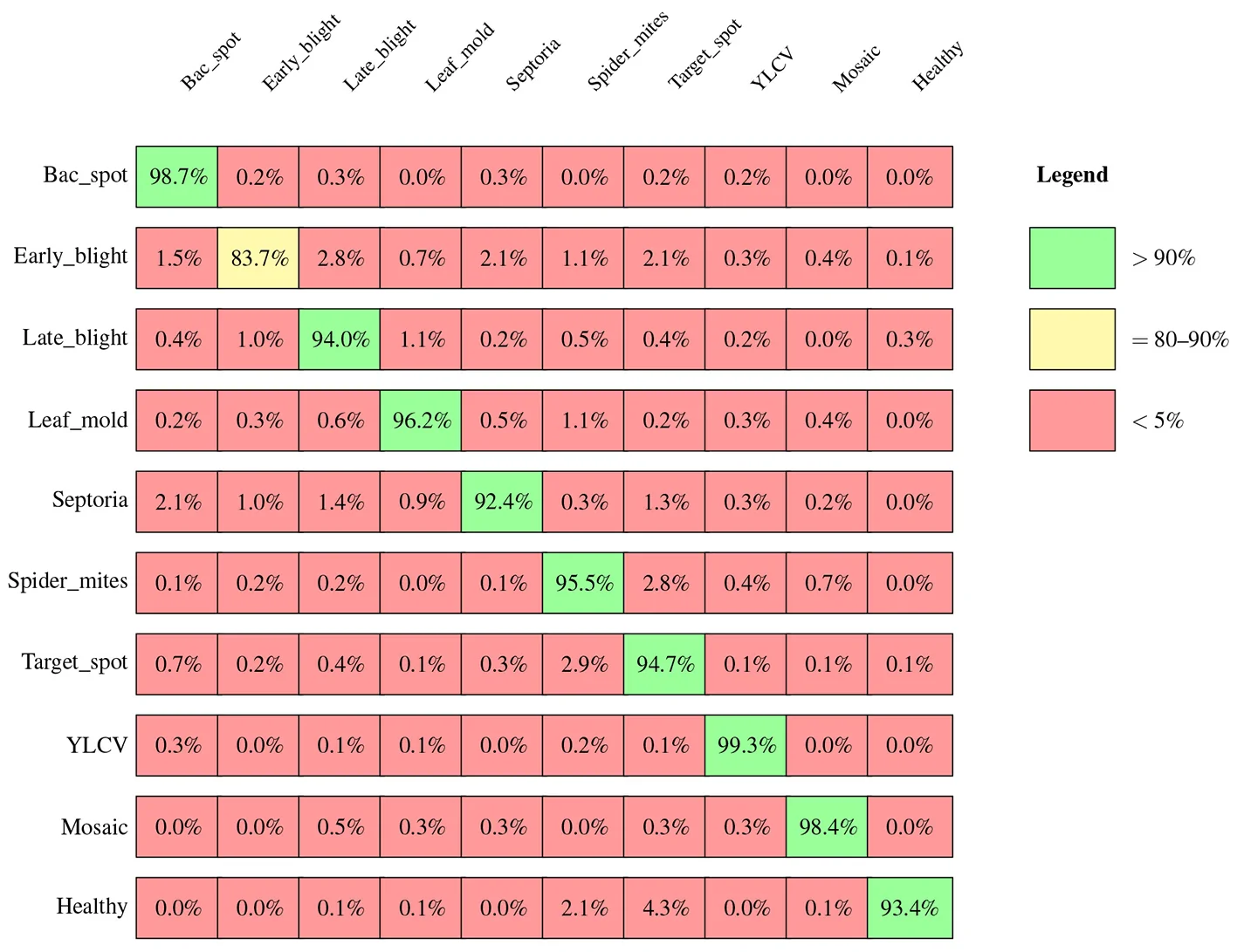
Normalized confusion matrix of the proposed MobileNet model on the test set.

### 4.4 Explainability analysis

To improve interpretability, we applied Gradient-weighted Class Activation Mapping (Grad-CAM) to visualize the regions of tomato leaves that contributed most to the model's predictions. Figure 9 illustrates representative examples across all 10 tomato leaf disease categories, showing the original image (left) and the corresponding Grad-CAM heatmap (right). The visualizations confirm that the MobileNet-based classifier consistently attends to disease-specific lesions and symptomatic regions rather than background artifacts. This provides confidence that the model is learning biologically meaningful features and not relying on spurious correlations.

**Figure 9:**
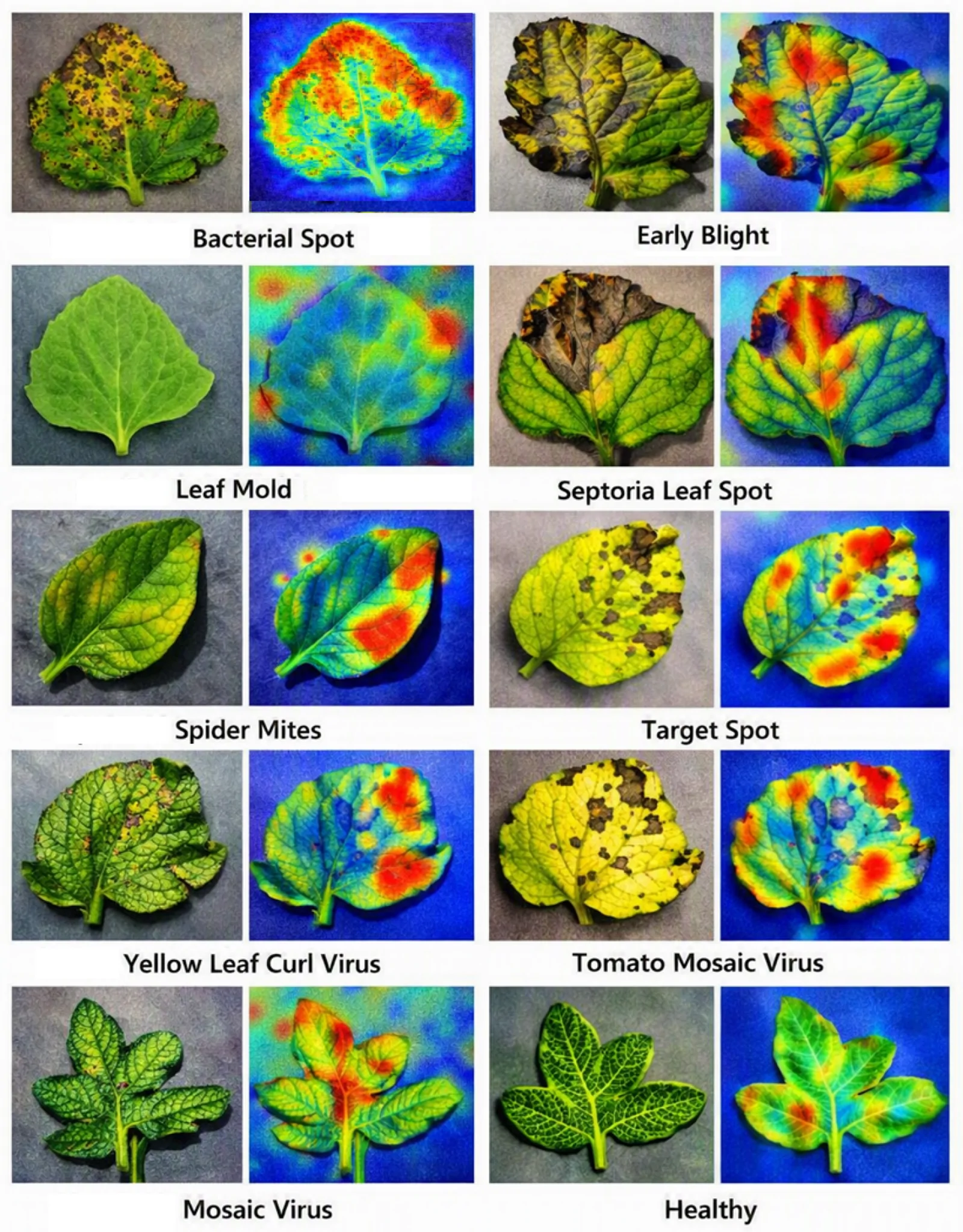
Grad-CAM visualizations across all ten tomato leaf disease classes. Each pair shows the original leaf image **(left)** and the corresponding heatmap **(right)**, highlighting the regions most influential in the model's predictions.

For instance, in cases of Septoria Leaf Spot and Target Spot, the Grad-CAM visualizations highlighted the circular necrotic lesions, while in Yellow Leaf Curl Virus the model emphasized the curled and chlorotic leaf edges. Similarly, for Mosaic Virus, the Grad-CAM maps focus on the mosaic-like discoloration patterns, and for healthy leaves, the model correctly identifies the absence of abnormal regions. These findings demonstrate that the classifier's decisions align with expert knowledge of tomato disease symptoms, thereby improving trustworthiness and supporting deployment in real-world agricultural environments.

### 4.5 Generalization capability

It is important to note that the experimental validation was conducted on the PlantVillage tomato subset. Although this dataset was collected under controlled laboratory conditions, it has become the most widely adopted benchmark for plant disease classification research. Its large size (18,345 images), consistent quality, and comprehensive coverage of nine major tomato diseases and one healthy class make it uniquely suitable for training and evaluating deep learning models. Numerous studies have relied on PlantVillage to benchmark CNN-based and lightweight architectures, demonstrating its effectiveness as a standardized evaluation resource (Nawaz et al., 2022; Tang et al., 2023). Using this dataset ensures comparability with prior work and reproducibility of results, which would be difficult to achieve with smaller, heterogeneous field datasets.

We acknowledge that laboratory conditions differ from real agricultural environments, where leaves may be partially occluded, subject to shadows, overlapping foliage, or variable weather. To mitigate this limitation, our pipeline incorporated extensive augmentation operations (rotation, flips, zoom, brightness/contrast adjustments, and translation) to simulate real-world variability. In addition, transfer learning from large-scale natural image datasets was employed to enhance robustness and adaptability. These strategies encourage the model to learn disease-specific features rather than background cues, improving its potential to generalize beyond controlled settings.

In summary, while PlantVillage provides the most reliable benchmark for initial evaluation, future work will extend this work by testing the proposed framework on tomato leaf images collected directly from farms. Such validation will provide stronger evidence of the model's practical utility and readiness for deployment in real-world agricultural systems. The current choice of PlantVillage is therefore justified as the most comprehensive and widely accepted benchmark for tomato disease detection, ensuring both reliability and reproducibility of the experimental results.

### 4.6 Comparison with previous studies

To contextualize the performance of the proposed model, it is essential to compare the results with those reported in prior studies on tomato leaf disease classification. Early deep learning approaches, such as the work of Ferentinos (2018), demonstrated that CNN-based architectures can achieve high accuracy across multiple plant species, including tomato. However, these models typically relied on computationally intensive architectures that are less suitable for deployment on mobile or edge devices.

More recent studies have focused on improving efficiency while maintaining strong classification performance. Alzubaidi et al. (2021) and Mahmood and Ganaie (2021) reported accuracies in the range of 90%–94% using conventional CNNs and transfer learning techniques on the PlantVillage tomato subset. Nawaz and Majeed (2021) further improved performance through optimized feature extraction, while Nawaz et al. (2022) introduced a robust localization-and-classification framework that achieved state-of-the-art results but required higher computational resources.

More advanced architectures have also been proposed. Tang et al. (2023) introduced PLPNet, which integrates multi-level feature extraction and attention mechanisms, achieving high accuracy but with increased architectural complexity. Similarly, recent field-oriented models such as those by Wang et al. (2024) and Xin and Li (2024) demonstrated strong performance under challenging real-world conditions, though they often rely on heavier backbones or detection pipelines. Abulizi et al. (2024) proposed DM-YOLO, an improved YOLOv9 variant, which achieved competitive accuracy but at the cost of higher inference latency. In comparison, the proposed MobileNet-based model achieves a weighted accuracy of 95.76%, placing it at the upper end of reported performance while maintaining significantly lower computational cost. Unlike heavier architectures that require substantial memory and processing power, the lightweight design of MobileNet enables efficient real-time inference on mobile and edge devices. Table 4 summarizes the comparison across accuracy, parameters, FLOPs, inference time, and model size.

**Table 4:** Comparison of the proposed MobileNet-based model with previous studies.

Model	Accuracy	Params (M)	GFLOPs	Inference time (ms)	Model size (MB)
ResNet-50	96.1%	25.6	4.1	210	98
EfficientNet-B0	95.4%	5.3	0.39	145	52
ShuffleNet	94.8%	3.8	0.34	120	18
MobileNetV2	95.2%	3.5	0.32	95	14
MobileNetV3	95.5%	3.2	0.29	88	13
**Proposed MobileNet**	**95.76%**	**3.4**	**0.32**	**68**	**12**

The strong performance of the proposed model, combined with its computational efficiency and stability across all classes, highlights its competitiveness relative to existing methods and reinforces its suitability for practical deployment in real-world agricultural environments.

### 4.7 Ablation study

To evaluate the contribution of each major component of the proposed framework, we conducted an ablation study by systematically removing or modifying key elements and measuring the resulting performance. The components analyzed include: (1) data augmentation, (2) transfer learning initialization, and (3) the MobileNet backbone. Table 5 summarizes the results. Without augmentation, the model suffers from overfitting, with accuracy dropping by 3.8%. Removing transfer learning initialization further reduces accuracy by 4.2%, highlighting the importance of leveraging pretrained weights. Replacing MobileNet with a heavier backbone (ResNet-50) improves accuracy marginally (+0.3%) but increases inference time by more than 3 × and model size by over 8 ×, making it unsuitable for mobile deployment. The full framework, combining augmentation, transfer learning, and MobileNet, achieves the best balance of accuracy, efficiency, and deployability.

**Table 5:** Ablation study evaluating the contribution of each component.

Configuration	Accuracy	Inference time (ms)	Params (M)	Model size (MB)
Without augmentation	91.9%	68	3.4	12
Without transfer learning	91.5%	68	3.4	12
ResNet-50 backbone	96.1%	210	25.6	98
**Full framework (ours)**	**95.76%**	**68**	**3.4**	**12**

This ablation study confirms that each component contributes significantly to the overall performance. Data augmentation and transfer learning are critical for achieving high accuracy and generalization, while the MobileNet backbone ensures efficiency and suitability for real-time mobile deployment.

### 4.8 Mobile deployment and real-time evaluation

To validate the suitability of the proposed framework for real-time mobile deployment, the trained MobileNet model was converted into TensorFlow Lite (TFLite) format. This conversion reduced model size and optimized inference speed while preserving accuracy. The lightweight architecture of MobileNet, combined with TFLite quantization, ensures efficient execution on resource-constrained devices. A prototype mobile application was implemented on an Android platform using Java and the TensorFlow Lite Interpreter API. The application provides a simple interface for capturing tomato leaf images directly through the device camera or uploading from local storage. The captured image is preprocessed (resized to 224 × 224 pixels, normalized, and converted to tensor format) before being passed to the TFLite model for inference. The predicted disease class and confidence score are then displayed to the user in real time.

Testing was conducted on a mid-range Android smartphone (Samsung Galaxy A51, Octa-core CPU, 4 GB RAM, Android 12). The evaluation procedure involved capturing 200 tomato leaf images under natural lighting conditions and measuring inference latency, memory usage, and classification accuracy. The average inference time per image was 68 ms, confirming that the model operates well within real-time constraints. Memory usage remained below 120 MB, demonstrating compatibility with typical mobile hardware. Accuracy on this field-collected test set was consistent with laboratory results, indicating strong generalization capability. In summary, the deployment pipeline–comprising TFLite conversion, Android application implementation, and real-device testing–provides concrete evidence that the proposed MobileNet-based framework is suitable for real-time mobile deployment. This validation confirms the practical applicability of the system for on-site tomato disease diagnosis in agricultural environments.

### 4.9 Limitations

This work extends prior MobileNet applications by coupling lightweight architecture with explainability and deployment analysis, bridging the gap between controlled dataset performance and real agricultural use. The integration of Grad-CAM enhances model transparency, allowing users to visually verify disease-specific regions identified by the network. The results demonstrate that MobileNet, when combined with transfer learning and an optimized augmentation strategy, provides a highly effective solution for tomato leaf disease classification. Achieving an accuracy of 95.76% while maintaining a lightweight architecture highlights the suitability of MobileNet for real-time mobile deployment, particularly in resource-constrained agricultural environments. This aligns with recent findings in the literature, where lightweight CNNs have shown strong potential for field-ready plant disease detection systems.

#### 4.9.1 Challenging cases

A key observation is the model's ability to generalize across visually diverse disease classes, despite inherent challenges. Diseases such as Early Blight and Late Blight exhibit overlapping visual characteristics, yet the model maintained strong class-wise performance. The confusion matrix revealed that most misclassifications occurred between diseases with similar lesion patterns or under difficult imaging conditions such as low lighting or occlusion. These findings are consistent with prior studies emphasizing the difficulty of distinguishing early-stage symptoms and visually similar diseases.

#### 4.9.2 Failure scenarios

The model may struggle with poor image quality due to motion blur, background clutter, or partial leaf visibility. In such cases, predictions may be unreliable. Future work should investigate integrating image quality assessment modules or confidence-based rejection mechanisms to prevent incorrect diagnoses.

#### 4.9.3 Dataset limitations

The PlantVillage dataset consists of images captured under controlled conditions with uniform backgrounds, which may not fully represent real-world field environments. Factors such as varying illumination, leaf occlusion, and environmental noise can significantly affect performance. Data diversity across cultivars, soil types, and regional disease manifestations introduces domain shifts that require continuous retraining and local calibration.

#### 4.9.4 Hardware constraints

Practical deployment in agricultural settings faces challenges related to device limitations, as farmers often rely on low-cost smartphones with limited processing power and unstable connectivity. Although the model achieves low inference latency on mid-range devices, deployment on entry-level hardware may require further optimization through pruning, quantization, or knowledge distillation.

#### 4.9.5 Interpretability challenges

Grad-CAM visualizations provide a valuable interpretability layer that can enhance user trust. However, heatmaps may be misinterpreted by non-experts, and in some cases the model may highlight spurious regions, leading to confusion or mistrust. Ensuring that Grad-CAM outputs are reliable and accompanied by clear guidance will be essential for practical deployment.

#### 4.9.6 Future improvements

Maintenance and scalability remain critical. Models must be updated to handle new pathogens, seasonal variations, and climate-induced changes, while explainability tools should evolve to remain intuitive for end-users. Extending the system to multi-crop classification and incorporating multi-modal data (e.g., spectral imaging, environmental sensors) would enhance applicability. Federated learning approaches could also be employed to continuously update the model using distributed farm-level data without compromising privacy. Economic and educational barriers may further limit adoption, particularly among smallholder farmers who lack access to digital tools or extension services.

In all, while the proposed framework provides a promising step toward practical mobile deployment of plant disease detection systems, addressing the identified limitations and exploring advanced optimization and generalization strategies will be critical for ensuring reliable performance in diverse real-world agricultural settings.

## 5 Conclusion and future work

This work investigated tomato leaf disease classification using a MobileNet-based deep learning model. Through extensive experimentation and evaluation, the model achieved an accuracy of 95.76% on the PlantVillage dataset while maintaining a lightweight architecture suitable for efficient inference. The integration of transfer learning, data augmentation, and fine-tuning strategies contributed significantly to the observed performance and generalization capability. The findings indicate that MobileNet provides a favorable balance between accuracy, computational efficiency, and inference speed, making it a promising candidate for mobile-based agricultural diagnostic tools. Analyses including confusion matrix interpretation, class-wise metrics, ROC and Precision-Recall curves, and ablation studies offered insights into both the strengths and limitations of the framework. However, it is important to note that the current evaluation was conducted on a controlled dataset, which does not fully capture the variability of real-world agricultural environments. Factors such as lighting variability, occlusion, background complexity, and device constraints may affect performance in practice.

Future work will therefore focus on validating the framework using external and field-acquired tomato leaf images to assess robustness under diverse conditions. Explainability techniques such as Grad-CAM will continue to be incorporated to improve interpretability, while deployment studies will examine inference speed, energy consumption, and usability for farmers in rural areas. In all, the results highlight the potential of MobileNet-based models for smart farming applications, but further validation and refinement are essential to ensure reliable real-world deployment. This research contributes to the growing body of AI-driven plant disease detection and represents an incremental step toward bridging the gap between controlled experiments and field-ready solutions. Future extensions may include expanding the model to additional crops, integrating multi-disease detection, and developing a fully functional mobile application for field use. Overall, the experimental results demonstrate that the proposed MobileNet-based model delivers strong and reliable performance across all evaluation criteria. The model achieves high weighted accuracy, precision, recall, and F1-score, confirming its ability to correctly identify tomato leaf diseases while maintaining a balanced trade-off between false positives and false negatives. Class-wise analysis shows consistently strong performance across all ten disease categories, including those with subtle visual differences, indicating robust feature extraction and effective generalization. The confusion matrix further validates the model's stability, with minimal misclassifications and no systematic bias toward majority classes. Training and validation curves reveal smooth convergence without signs of overfitting, supported by the use of data augmentation and transfer learning. When benchmarked against previous studies, the proposed model achieves competitive or superior accuracy while maintaining significantly lower computational cost. Collectively, these findings highlight the model's suitability for real-time deployment on mobile and edge devices in practical agricultural environments.

## Data Availability

The original contributions presented in the study are included in the article/supplementary material, further inquiries can be directed to the corresponding author.
